# Clinical and surgical aspects of congenital lobar over-inflation: a single center retrospective study

**DOI:** 10.1186/s13019-020-01145-8

**Published:** 2020-05-19

**Authors:** Mohamed Abdel-Bary, Mohamed Abdel-Naser, Ahmed Okasha, Mohammed Zaki, Khaled Abdel-Baseer

**Affiliations:** 1 Department of Cardiothoracic Surgery, Qena Faculty of Medicine, South Valley University, Safaga Road, Qena, 83523 Egypt; 2 Department of Anaesthesia and ICU, Assiut Faculty of Medicine, Assiut University, Assiut, Egypt; 3 Department of Radiology, Qena Faculty of Medicine, South Valley University, Qena, Egypt; 4 Department of Radiology, Sohag Faculty of Medicine, Sohag University, Sohag, Egypt; 5 Department of Pediatrics, Qena Faculty of Medicine, South Valley University, Qena, Egypt

**Keywords:** Congenital lobar overinflation, Lobectomy, Neonatal respiratory distress

## Abstract

**Background:**

Congenital lobar overinflation (CLOI) is one of the most important causes of infantile respiratory distress (RD). We aim to evaluate our experience in CLOI management emphasizing on clinical features, diagnostic modalities, surgery and outcomes.

**Methods:**

This is a retrospective study for all CLOI cases undergoing surgical management at Qena University Hospital. Demographic data, clinical data, radiographic findings, surgery and postoperative follow-up were reviewed.

**Results:**

A total of 37 neonates and infants with CLOI were presented to our center between January 2015 and January 2019; their mean age was 111.43 ± 65.19 days and 22 were males. All cases presented with RD; and cyanosis in 19 cases. 15 cases presented with recurrent pneumonia and fever. Diminished breath sounds on the affected side and wheezes were the main clinical findings in 30 and 22 cases respectively. On CXR, emphysema was detected in all cases. A confirmatory CT chest was done for all cases. Left upper lobe was affected in 23 cases, right middle lobe in 7 and right upper lobe in 7 cases.

Lobectomy was done in thirty-one cases; their mean age at surgery was 147.58 ± 81.49 days and 19 were males. Postoperative complications were noted in 5 cases and postoperative ventilation was required for 2 of them. No morbidity or mortality was reported. The follow-up duration ranged from 3 months to 1 year and all patients were doing well except one case that lost follow up after 3 months.

**Conclusion:**

CLOI is a rare bronchopulmonary malformation that requires a high index of clinical suspicion, especially in persistent and recurrent infantile RD. CT chest is the most useful diagnostic modality. Early management of CLOI improves outcome and avoid life-threatening complications. Surgical management is the treatment of choice in our center without recorded mortality.

## Introduction

Congenital lobar over-inflation (CLOI) is one of the rare bronchopulmonary malformations [1]. Till now, its exact etiology isn't known. But many authors reported that; it occurs due to bronchoalveolar congenital anomalies without external bronchial compression [2]. Also, it is well known by a developing over-inflation of a pulmonary lobe, compression of the healthy lung tissue with the mediastinum and trans mediastinal herniation of the over-inflated lobe. Thus, the oxygenation and venous return are impaired; that leads to varying degrees of respiratory distress (RD) and hypotension [3, 4]. Nearly half of the infants symptomatize at birth, while the others mostly manifest later. It is documented that CLOI may be associated with other systems anomalies especially cardiac in 20% of cases [5].

It most frequently affects the left upper lobe (LUL) followed by right middle lobe (RML). However, acquired lobar over-inflation (ALOI) may occur due to external bronchial compression or endobronchial lesions or a foreign body; they lead to air entrapment and hyperinflation. So, bronchoscopy is needed to exclude or manage these cases [6]. Sometimes, CLOI diagnosis represents a challenge to pediatricians. So, its initial diagnosis can save baby life. Moreover, RD is the most frequent clinical scenario in neonates may be associated with wheezes, cough, and cyanosis; that may be aggravated during feeding or crying. Also, they may come with repeated chest infections and failure to thrive. The condition might be confused with other causes of neonatal RD like pneumonia and pneumothorax [7].

Diagnosis is suspected clinically and with chest X-ray (CXR); that can be confirmed by chest computed tomography (CT). Radiologically, it's characterized by over-aeration of the affected lobes with a mediastinal shift [8].

Lobectomy is the classically accepted treatment for CLOI. However, conservation for cases with mild symptoms was reported [9].

We aim to present our clinical and surgical experience for the management of CLOI in infants submitted to lobectomy with emphasizing on natural history and postoperative outcomes.

## Methods

### Study population

This is a retrospective study investigated 37 neonates and infants with CLOI underwent surgical management at pediatrics and cardiothoracic surgery departments, Qena University Hospital, Egypt between 2015 and 2019. The indication for surgical resection was the clinically severe RD; that was confirmed radiologically.

Preoperative clinical data included age at presentation, gender, clinical findings, preoperative imaging (CXR and CT chest), the affected lobes, bronchoscopic findings (when bronchoscopy was performed) and other malformations.

Overall, lobectomy was done for 31 cases of them; because three cases died due to pneumonia and three cases lost to follow up preoperatively.

The operation was included. Postoperative data included the age at surgery, improvement of the symptoms; re-expansion of the remaining lung; radiographic imaging; complications; total length of hospital stay; the need for mechanical ventilation, and follow-up duration. The CLOI diagnosis was confirmed pathologically. Postoperative morbidity and mortality were reported. All the cases completed the follow-up, it was done on regular outpatient clinic visits after discharge.

The study conforms to the ethical standards of the Helsinki Declaration and approval was obtained from the institutional ethics committee of Qena Faculty of Medicine.

### Anaesthetic and operative approaches

**Fig. 1:**
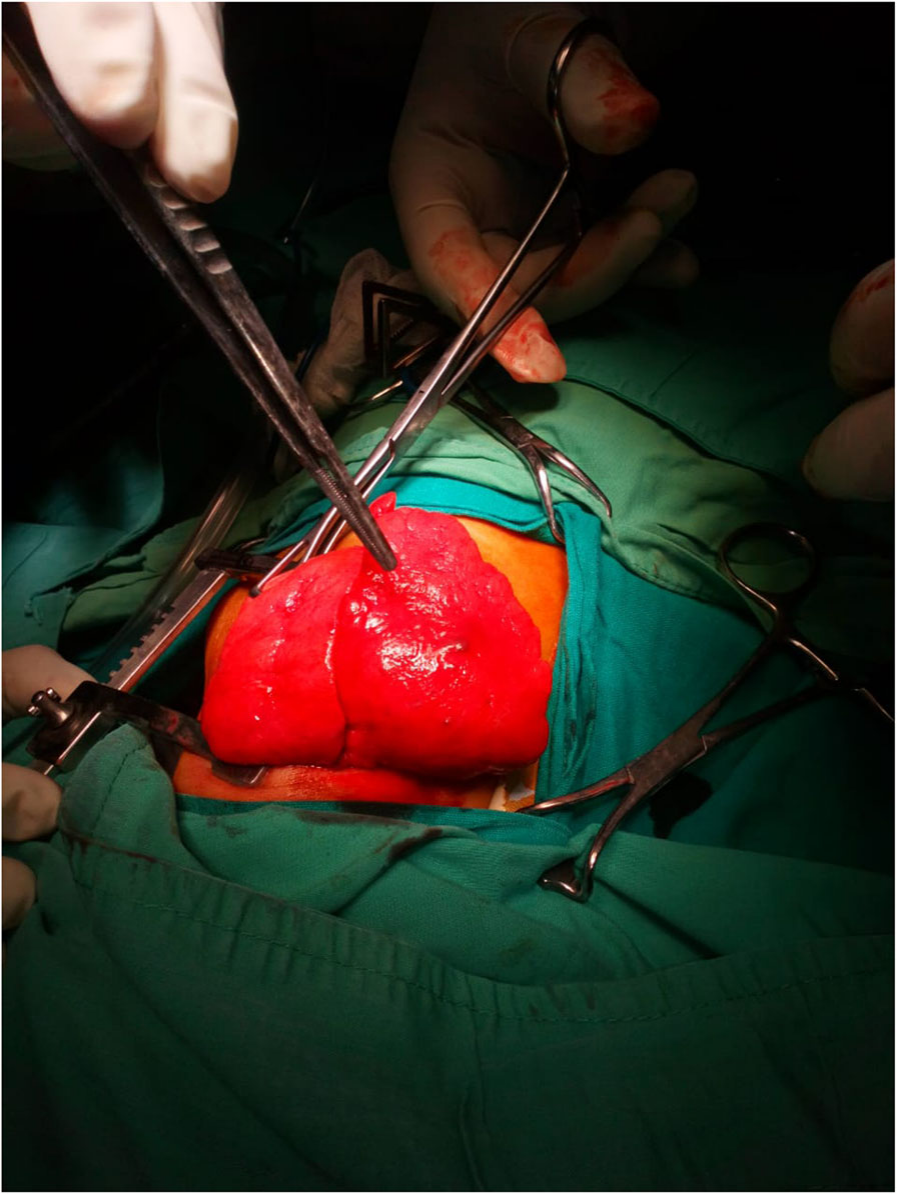
The emphysematous left upper lobe popped out through the thoracotomy incision

The cases were kept fasting according to guidelines. In the operative theatre, the ideal monitoring was instituted. Pre-oxygenation with 100% oxygen was done. Anesthesia induction was started with 1%-8% sevoflurane. Proper sized uncuffed single-lumen endotracheal tube was used. Proper anaesthesia with gently assisted ventilation was maintained. The lobectomy was done by the same surgeon, and he was ready for emergency intervention if needed. The patients were placed in lateral recumbent position; the diseased side up and fixed with adhesive plaster and a pad under the chest. Anesthesia was maintained with 2%-3% sevoflurane [10]. A muscle-sparing posterolateral thoracotomy incision was done. The thoracic cavity was entered via the fourth or fifth intercostal space. It was maintained opened by a self-retaining rib spreader. Gentle assisted manual ventilation was continued until the emphysematous lobe popped out through the thoracotomy incision (Fig. 1); to relieve the compression and improve the oxygenation. Cisatracurium 0.1-0.2 mg/kg IV mg was then administered. Positive pressure ventilation was initiated, using pressure-controlled mode keeping peak inspiratory pressure around 20 cmH2O. Anatomical lobectomy was performed. Intraoperative analgesia was maintained with fentanyl 2 microgram/kg given intermittently.

Post lobectomy, manual ventilation was used to expand the compressed residual lung to occupy the thoracic cavity. Also, the saline test was used to test for the presence of air leaks before closure. Two intercostal tubes (ICT) were inserted. The intercostal nerve block was given with 3 ml of 0.25% bupivacaine. The chest was closed in layers. Intravenous ringer lactate and blood were used if needed. Residual neuromuscular blockade was reversed with atropine 20 mcg/kg and neostigmine 30-70 mcg/kg IV. Extubation was done on the table after regaining of airway reflexes and adequate spontaneous breathing. Then the cases were transferred to the pediatric intensive care unit (ICU) for observation [10]. The follow-up CXR was ordered on the first day postoperative. The ICT was removed; when the lung is fully expanded, no air leak, minimal oscillation in ICT and no bleeding.

### Statistical analysis

Continuous variables were expressed as mean ± standard deviation, and categorical variables were expressed as frequency.

## Results

### Preoperative data

Thirty-seven CLOI cases were presented to our hospital. Their mean age was 111.43 ±65.19 days and 22 (59%) were males. All cases presented with RD (tachypnea, dyspnea); and cyanosis in 19 cases (51%). Fifteen cases (38%) presented with recurrent pneumonia associated with fever; and cough in 12 cases (32%). Moreover, diminished breath sounds on the affected side and wheezes were the main clinical findings in 30 (81%) and 22 (59%) cases respectively.

**Fig. 2:**
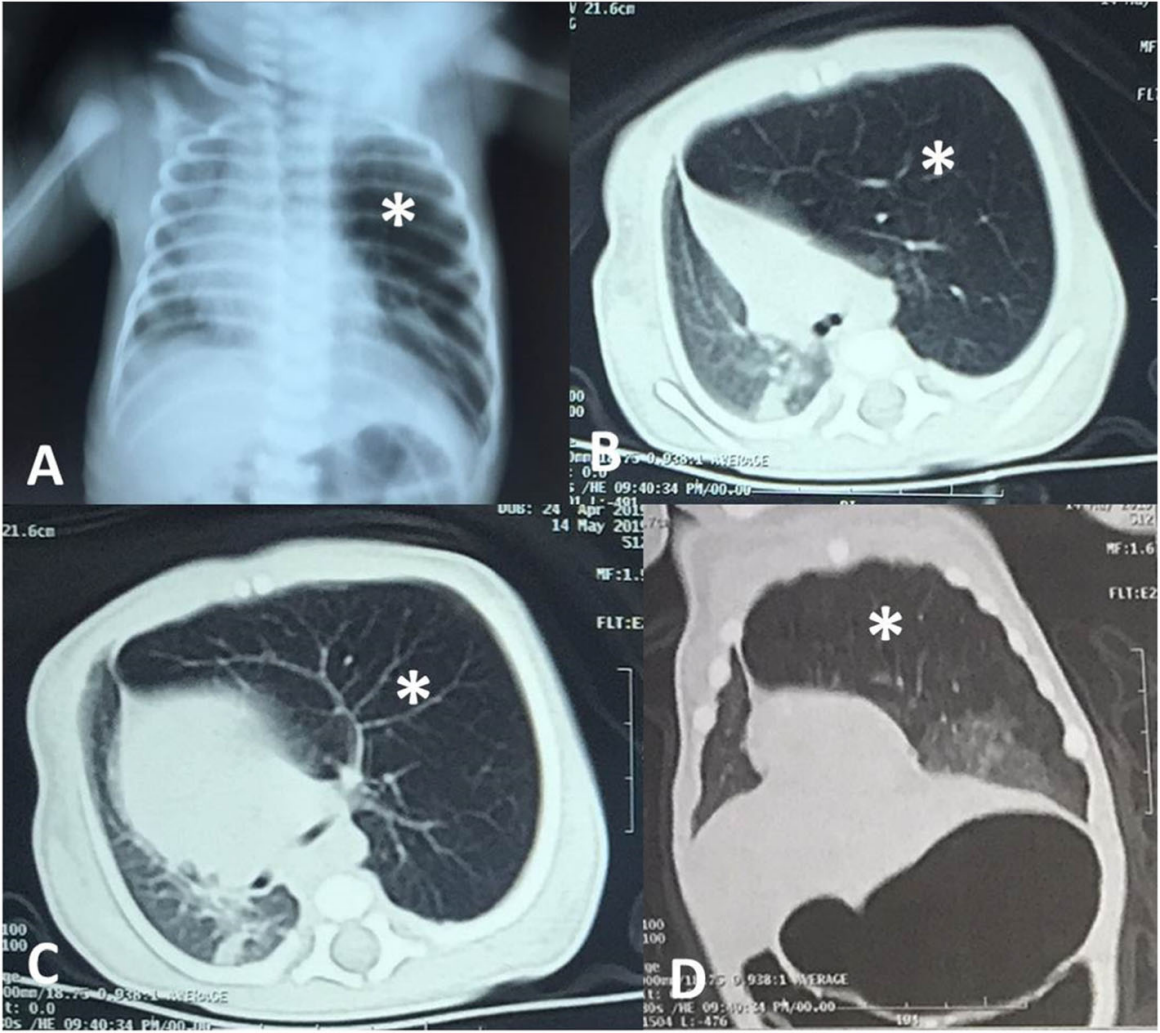
Male infant 5 weeks old has congenital left upper lobe over inflation. **a** Chest X-Ray, **b**&**c** Axial CT lung window and **d** Oblique coronal CT lung window showing the characteristic radiological findings of congenital lobar overinflation (*) and mediastinal shift. Crossing of the overinflated lobe to the other side is also noted

**Fig. 3:**
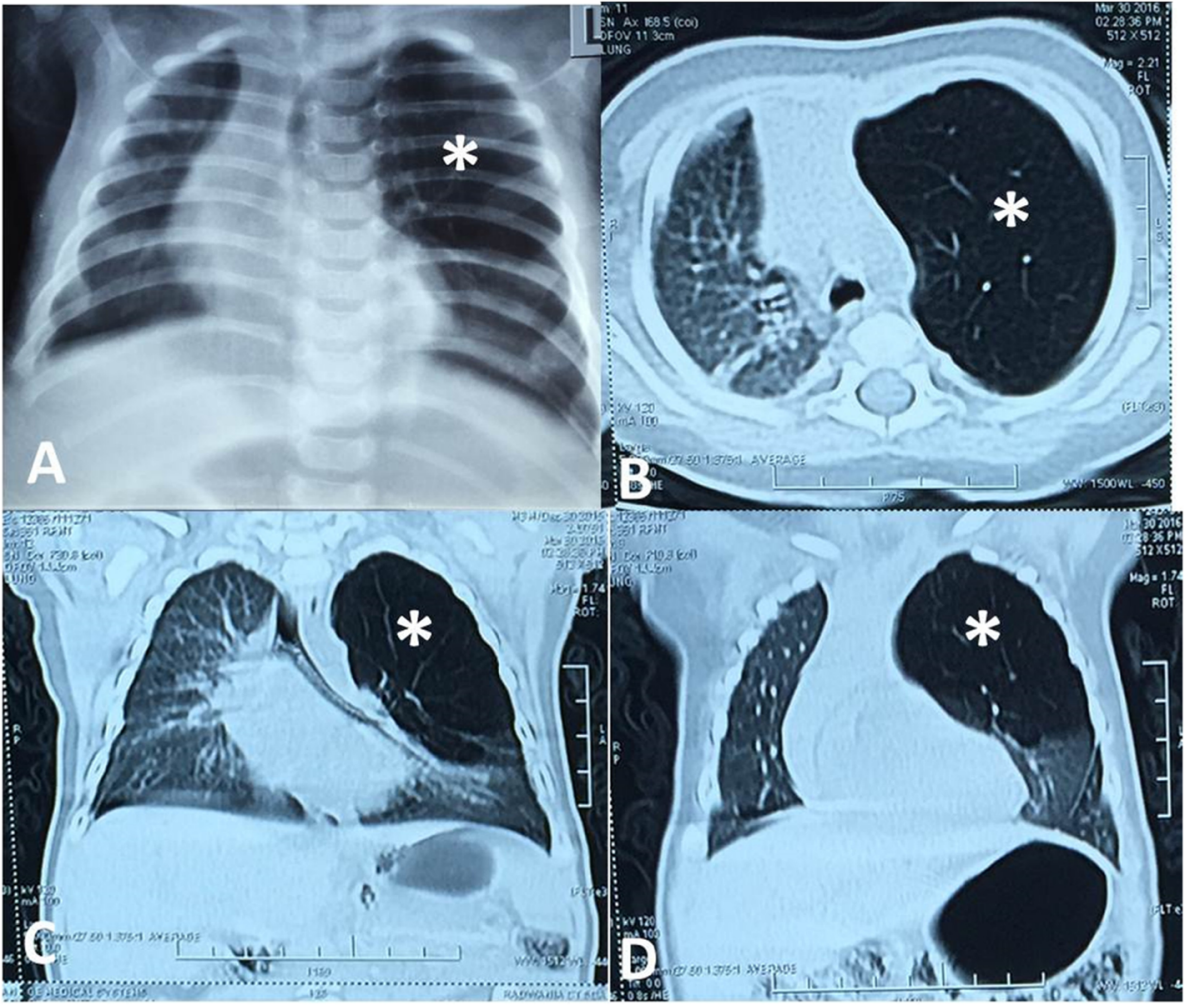
Male infant of 3 months-old has congenital left upper lobe overinflation. **a** Chest X-Ray, **b** Axial CT lung window and **c**&**d** Coronal CT lung window showing left upper congenital lobar overinflation (*) and mild mediastinal shift

**Fig. 4:**
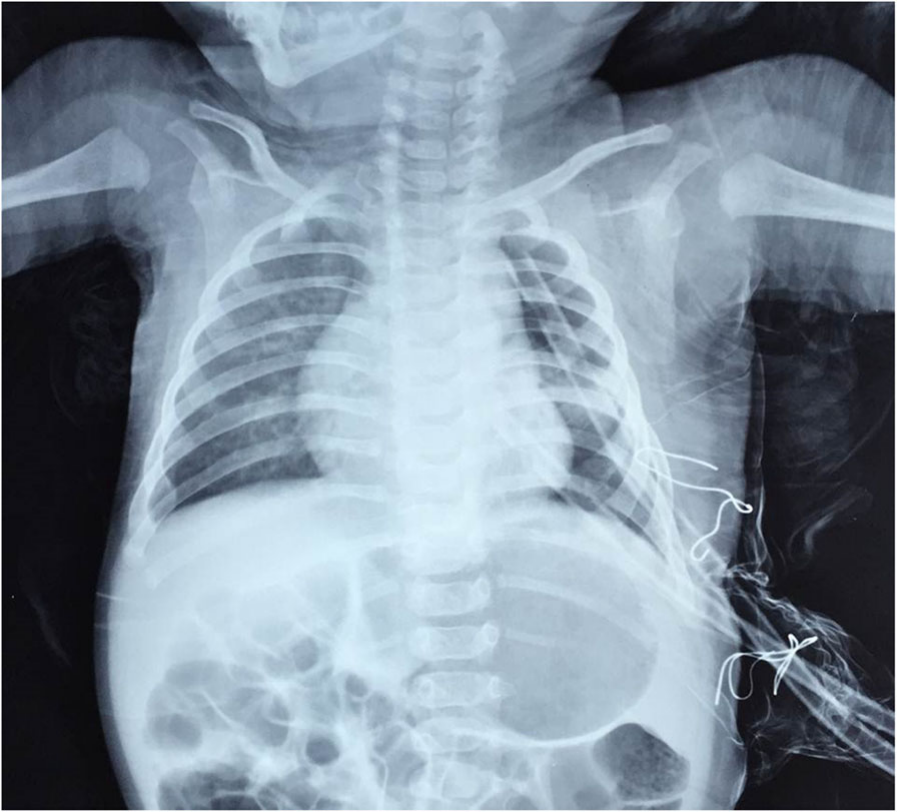
Post-operative Chest X-Ray for a patient with left upper lobe overinflation. It shows reduced left lung volume without mediastinal shift. Post-operative intercostal tubes are noted

On CXR, emphysema was detected in all cases; while a mediastinal shift to the opposite lung and atelectasis were observed in 18 (49%) and 10 (27%) cases respectively. A confirmatory CT chest was done for all cases, and the findings consisted of hyperinflation of the affected lobe, contralateral herniation of the affected lobe through the anterior mediastinum, mediastinal shift, and collapse of the healthy lobes due to compression (Figs. 2, 3 and 4). CLOI was noticed most frequently in the left upper lobe (LUL) 23 cases (62%); followed by the right upper lobe 7 cases (19%) and right middle lobe 7 cases (19%). Associated cardiac anomalies were observed in 9 cases (24%); they were managed later on. Cleft lip and palate were found in two cases (5%); they were managed in another setting. Bronchoscopy (rigid & fiberoptic) was performed in 6 cases (16%). In two cases we found marked bronchomalacia with slit-like ostium of the LUL bronchus; while the others reported nonspecific findings (Table 1).

**Table 1:** Preoperative demographic, clinical and radiographic features at diagnosis

Variable	Number (37)	Percentage%
Age at presentation (days)	111.43 ± 65.19	
**Gender**		
**Male**	22	59
**Female**	15	41
Symptoms		
Cyanosis	19	51
Tachypnea	37	100
Dyspnea	37	100
Cough	12	32
Signs		
Diminished breath sounds on the affected side	30	81
Fever	15	38
Wheezes	22	59
Pneumonia		
Recurrent pneumonia as a presenting diagnosis	15	38
Affected lobe		
Left upper lobe (LUL)	23	62
Right upper lobe (RUL)	7	19
Right middle lobe (RML)	7	19
Chest x ray		
Emphysema	37	100
Shift to opposite side	18	49
Atelectasis	10	27
Bronchoscopy (rigid & fiberoptic)	6	16
CT chest		
Emphysema	37	100
Shift to opposite side	21	68
Atelectasis	8	22
Associated Anomalies		
Cardiac	9	24
Cleft lip & palate	2	5

### Operative data

Lobectomy was done in thirty-one cases. Their mean age at surgery was 147.58 ± 81.49 days and 19 (61%) were males. The time gap between the diagnosis and the surgery ranged from 2 weeks to 3 months. The mean operation time was 133.83 ± 47.64 min. The blood loss was minimal in all cases. All cases recovered from anesthesia and extubated on the table; except for 2 cases (6%) were transferred to ICU and mechanically ventilated; they were weaned successively within 4 to 7 days (Table 2).

**Table 2:** Demographic, clinical and radiographic features on operative data and postoperative follow up

Variable	Number (31)	Percentage%
Age at surgery (days) mean ± SD	147.58 ± 81.49	
Gender		
Male	19	61
Female	12	39
Symptoms		
Cyanosis	0	0
Tachypnea	0	0
Dyspnea	2	6
Cough	3	10
Signs		
Decreased breath sounds	0	0
Wheezes	1	3
CXR		
Normal	29	94
Shift to opposite side	0	0
Atelectasis	0	0
Hypertranslucency	2	6
Postoperative ventilation	2	6
Operative time: mean ± SD (min)	133.83 ± 47.64	
Postoperative complications		
Pneumonia	3	10
Wound infection	1	3
Pneumothorax	1	3
Late outcome		
Well	30	97
Lost follow up	1	3

### Postoperative data

Marvellous resolution of RD symptoms was noticed in all cases; however, cough, dyspnea and wheezes were detected in 3 (10%), 2 (6%) and one case (3%) respectively. Follow up CXR appeared about to be normal in 29 cases (94%) (Fig. 4); except hypertranslucency in two cases (6%). CLOI diagnosis was confirmed by pathological examination of the resected lobe in all cases.

ICT removal and discharge were done within up to 5 and 7 days after surgery, respectively in non-complicated cases. And up to 15 days in complicated cases.

In 5 cases (16%) we reported postoperative complications. Pneumonia occurred in 3 cases (10%) that required a change of antibiotics. Wound infection was reported in one case (3%); it was managed by daily dressing and antibiotics. One case (3%) suffered from pneumothorax that needed ICT reinsertion. The ICTs were removed after (3-7) days post-operatively in all cases.

All cases experienced well-being through 12 months follow up at outpatient postoperatively; except one case (3%) lost to follow up after 3 months. No morbidity or mortality was reported (Table 2).

## Discussion

Congenital lobar over-inflation is an infrequent entity, but an important cause of neonatal RD. It is characterized by over-inflation of one or more lung lobes with preserved structure after birth and can be diagnosed prenatally by ultrasound [11–13]. In agreement with other studies, we found that CLOI is more common in males [14].

Regarding lobar affection, the LUL was the most affected followed by RUL and RML. This agreed with the literature; that the LUL is most commonly affected unilaterally followed by RUL and RML in a similar approximate percentage of anatomical distribution [8, 15–17]. Even though lower lobe affection and bilateral lobe affection are extremely rare and not detected in this study; they have been recorded by others [18–20]. The contradiction between unique studies in rates of CLOI lobes affection may be due to the distinction between the number of patients and variable duration of these studies. The exact etiology of CLOI could not be detected. However, the acquired causes of lobar over-inflation should be excluded. It may occur due to external compression by cyst, vessel, mass or lymphadenopathy; or internally by endobronchial lesions or foreign bodies [21, 22].

Neonatal and infantile RD were the presenting symptoms within the first 6 months in all cases; this was consistent with those reported in the literature [23]. However, Man et al. reported a delayed presentation in children 5 years age [18]. In agreement with Cataneo et al. [14], we found recurrent chest infection as a common presentation in a considerable number of cases and if combined with respiratory distress may delay the diagnosis especially in older infants.

It is well documented that CLOI can be diagnosed prenatally by fetal ultrasound, but most commonly missed diagnosis occurs and is detected postnatally in neonates when progressive hyperinflation induces manifestation by compression of the remaining ipsilateral and the contralateral lung leading to cardiopulmonary compromise [21, 24–26]. In our study, CLOI was apparent on routine CXR in all cases and atelectasis was an associated finding. However, CT chest was used to verify radiographic findings and rule out other bronchopulmonary or vascular anomalies.

It was reported that CLOI can be accompanied by cardiac anomalies in 14–20% of cases and by other anomalies in a lesser extent [5]. we found 24% of our cases with CHD and for this reason, Echocardiographic examination should be done for all CLOI cases to exclude cardiac anomalies before surgery.

Preoperatively, bronchoscopy (rigid & fiberoptic) was performed to exclude the causes of ALOI. Thus, we can overcome the wrong decision making. It was done in 6 cases (16%). In two cases we found marked bronchomalacia with the slit-like ostium of the LUL bronchus; while the others reported nonspecific findings. Also, this agreed with Karnak et al. [12].

For the cases with early presentation instantly after birth, some authors recommended perioperative preconditioning with high-frequency ventilation, selective intubation, or bronchoscopic decompression of the over-inflated lobe [27–29]. However, we didn’t use any of these maneuvers in this study.

Many authors reported that the classical management of CLOI is lobectomy [14, 19, 30, 31]. Thirty-one of our cases were managed by surgical lobectomy. Conservation wasn’t done in our cases.

Interestingly, there is a debate about CLOI management surgery versus conservative. Some authors considered the nonoperative approach in mild symptomatic cases [9, 19, 32]. However, regarding the extent of resection, some surgeons reported that advantages of CLOI conservative surgery by segmental lung resection rather than anatomical lobectomy [4, 33–36].

We operated all our cases with a conventional muscle-sparing posterolateral thoracotomy. Despite the advantages of thoracoscopic lung resection over traditional surgery in infants without increased risk of morbidity with a favourable long-term outcome; Rahman et al. found this option difficult in CLOI management. Also, Bawazir reported a conversion to open thoracotomy in his study [16]. The explanation for this; the over-inflated lobe occupies all the hemithorax that leads to difficulty in the creation of an operation plane. If we tried to insufflate gas for artificial pneumothorax, this may aggravate the compression and hypoxia [16, 36–38]. Mean a while, Calzolari F and Zoeller C reported a major complication after thoracoscopic lobectomy in infants is associated with major complications [39, 40].

In the current study, postoperative complications were found in 16% of cases; three cases were suffering from pneumonia, one case with pneumothorax and one case with wound infection. Mechanical ventilation was necessitated for two cases of them due to respiratory failure and weaned successively within 5 to 7 days without reported deaths. By previous studies, Surgical removal was considered the most common treatment choice with an operative mortality rate of about 3 to 7% [31] mortality was related more to the small body weight that adds challenge to both surgeons and the postoperative care unit in addition to associated congenital lesions, especially in the heart. Nazem et al. detected postoperative high mortality of approximately 13% in their study that included 30 cases of CLOI and reported that the number of affected lobes and base deficit at presentation were related to this unusual high mortality rate [41]. The most important limitation of our study is the relatively small number of cases and the retrospective nature of the study design.

## Conclusion

CLOI is a rare congenital lung malformation that requires ahigh index of clinical suspicion, especially in persistent and recurrent infantile RD. CT chest is the most useful diagnostic modality. Early management of CLOI improves outcome and avoid life-threatening complications. Surgical management is the treatment of choice in our center without recorded mortality.

## Data Availability

The datasets used or analyzed during the current study are available from the corresponding author on reasonable request.

## Abbreviations

CLOI: Congenital lobar over-inflation
ALOI: Acquired lobar over-inflation
CT: Computed tomography
CXR: Chest X-ray
ICT: Intercostal tube
ICU: Intensive Care Unit
RD: Respiratory distress
LUL: Left upper lobe
RML: Right middle lobe

## References

[1] Al-Salem AH, Gyamfi YA, Grant CS (1990). Congenital lobar emphysema. Can J Anaesth.

[2] Hangul M, Kose M, Demir OF (2019). Congenital lobar emphysema: diagnosis and treatment options. Int J Chron Obstruct Pulmon Dis.

[3] Roberts P, Holland A, Halliday R, Arbuckle SM, Cass D (2002). Congenital lobar emphysema: like father like son. J Pediatr Surg.

[4] Paramalingam S, Parkinson E, Sellars M, Diaz-Cano S, Nicolaides KH, Davenport M (2010). Congenital segmental emphysema: an evolving lesion. Eur J Pediatr Surg.

[5] Mendeloff EN (2004). Sequestrations, congenital cystic adenomatoid malformations, and congenital lobar emphysema. Semin Thorac Cardiovasc Surg.

[6] Moideen I, Nair SG, Cherian A, Rao SG (2006). Congenital lobar emphysema associated with congenital heart disease. J Cardiothorac Vasc Anesth.

[7] Prabhu M, Joseph TT (2012). Congenital lobar emphysema: challenges in diagnosis and ventilation. Anesth Essays Res.

[8] Thacker PG, Rao AG, Hill JG, Lee EY (2014). Congenital lung anomalies in children and adults current concepts and imaging findings. Radiol Clin N Am.

[9] Mei-Zahav M, Konen O, Manson D, Langer JC (2006). Is congenital lobar emphysema a surgical disease?. J Pediatr Surg.

[10] Saini S, Prakash S, Rajeev M, Girdhar KK (2017). Congenital lobar emphysema: anaesthetic challenges and review of literature. J Clin Diagn Res.

[11] Costanzo S, Filisett IC, Vella C, Rustico M, Fontana P (2016). Pulmonary malformations: predictors of neonatal respiratory distress and early surgery. J. Neonatal Surg.

[12] Karnak I, Senocak ME, Ciftci AO, Buyukpamukcu N (1999). Congenital lobar emphysema: diagnostic and therapeutic considerations. J Pediatr Surg.

[13] Tempe DK, Virmani S, Javetkar S, Banerjee A, Puri SK, Datt V (2010). Congenital lobar emphysema: pitfalls and management. Ann Card Anaesth.

[14] Cataneo DC, Rodrigues OR, Hasimoto EN, Schmidt AF, Cataneo AJ (2013). Congenital lobar emphysema: 30-year case series in two university hospitals. J Bras Pneumol.

[15] Doğan R, Demircin M, Sarıgül A, Paşaoğlu İ, Göçmen A, Bozer AY (1997). Surgical treatment of congenital lobar emphysema. Turk J Pediatr.

[16] Bawazir OA (2020). Congenital lobar emphysema: thoracotomy versus minimally invasive surgery. Ann Thorac Med.

[17] Wasilewska E, Lee EY, Eisenberg RL (2012). Unilateral hyperlucent lung in children. AJR Am J Roentgenol.

[18] Man DW, Hamdy MH, Hendry GM, Bisset WH, Forfar JO (1983). Congenital lobar emphysema: problems in diagnosis and management. Arch Dis Child.

[19] Perea L, Blinman T, Piccione J, Laje P (2017). Bilateral congenital lobar emphysema: staged management. J Pediatr Surg.

[20] Berrocal T, Madrid C, Novo S (2004). Congenital anomalies of the tracheobronchial tree, lung, and mediastinum: embryology, radiology, and pathology. Radiographics.

[21] Thakral CL, Maji DC, Sajwani MJ (2001). Congenital lobar emphysema: experience with 21 cases. Pediatr Surg Int.

[22] Lee EY, Dorkin H, Vargas SO (2011). Congenital pulmonary malformations in pediatric patients: review and update on etiology, classification, and imaging findings. Radiol Clin N Am.

[23] Kunisaki SM, Saito JM, Fallat ME, St. Peter SD, Kim AG, Johnson KN (2019). Current operative management of congenital lobar emphysema in children: a report from the Midwest pediatric surgery consortium. J Pediatr Surg.

[24] Ozcelik U, Gocmen A, Kiper N, Dogru D, Dilber E, Yalcin EG (2003). Congenital lobar emphysema: evaluation and long-term follow-up of thirty cases at a single center. Pediatr Pulmonol.

[25] Olutoye OO, Coleman BG, Hubbard AM, Adzick NS (2000). Prenatal diagnosis and management of congenital lobar emphysema. J Pediatr Surg.

[26] Correia-Pinto J, Gonzaga S, Huang Y, Rottier R (2010). Congenital lung lesions--underlying molecular mechanisms. Semin Pediatr Surg.

[27] Goto H, Boozalis ST, Benson KT, Arakawa K (1987). High-frequency jet ventilation for resection of congenital lobar emphysema. Anesth Analg.

[28] Glenski JA, Thibeault DW, Hall FK, Hall RT, Germann DR (1986). Selective bronchial intubation in infants with lobar emphysema: indications, complications, and long-term outcome. Am J Perinatol.

[29] Phillipos EZ, Libsekal K (1998). Flexible bronchoscopy in the management of congenital lobar emphysema in the neonate. Can Resp J.

[30] Costa AS, Perfeito JAJ, Forte V (2008). Surgical treatment of 60 patients with pulmonary malformations: what have we learned?. J Bras Pneumonol.

[31] Andrade CF, da Costa Ferreira HP, Fischer GB (2011). Congenital lung malformations. J Bras Pneumol.

[32] Ceran S, Altuntas B, Sunam GS, Bulut I (2010). Congenital lobar emphysema: is surgery routinely necessary?. Afr J Paediatr Surg.

[33] Krivchenya DU, Rudenko EO, Dubrovin AG (2013). Congenital emphysema in children: segmental lung resection as an alternative to lobectomy. J Pediatr Surg.

[34] Lilly JR, Wesenberg RL, Shikes RH (1976). Segmental lung resection in the first year of life. Ann Thorac Surg.

[35] Rahman N, Lakhoo K (2009). Comparison between open and thoracoscopic resection of congenital lung lesions. J Pediatr Surg.

[36] Bagrodia N, Cassel S, Liao J, Pitcher G, Shilyansky J (2014). Segmental resection for the treatment of congenital pulmonary malformations. J Pediatr Surg.

[37] Diamond IR, Herrera P, Langer JC, Peter CW, Kim PCW (2007). Thoracoscopic versus open resection of congenital lung lesions: a case-matched study. J Pediatr Surg.

[38] Moyer J, Lee H, Vu L (2017). Thoracoscopic lobectomy for congenital lung lesions. Clin Perinatol.

[39] Calzolari F, Braguglia A, Valfrè L, Dotta A, Bagolan P, Morini F (2016). Outcome of infants operated on for congenital pulmonary malformations. Pediatr Pulmonol.

[40] Zoeller C, Ure BM, Dingemann J (2018). Perioperative complications of video-assisted thoracoscopic pulmonary procedures in neonatesand infants. Eur J Pediatr Surg.

[41] Nazem M, Hosseinpour M (2010). Evaluation of early and late complications in patients with congenital lobar emphysema: a 12 year experience. Afr J Paediatr Surg.

